# Identification of Profound Metabolic Alterations in Human Dendritic Cells by Progesterone Through Integrated Bioinformatics Analysis

**DOI:** 10.3389/fimmu.2021.806110

**Published:** 2021-12-17

**Authors:** Sainan Zhang, Su Liu, Ling Hong, Xiaohui Wang, Lianghui Diao, Songchen Cai, Tailang Yin, Yong Zeng

**Affiliations:** ^1^ Reproductive Medical Center, Renmin Hospital of Wuhan University and Hubei Clinic Research Center for Assisted Reproductive Technology and Embryonic Development, Wuhan, China; ^2^ Shenzhen Key Laboratory of Reproductive Immunology for Peri-implantation, Shenzhen Zhongshan Institute for Reproduction and Genetics, Shenzhen Zhongshan Urology Hospital, Shenzhen, China

**Keywords:** progesterone, dendritic cells, RNA-seq, OXPHOS, fatty acid metabolism

## Abstract

Maintaining the homeostasis of the decidual immune microenvironment at the maternal-fetal interface is essential for reproductive success. Dendritic cells (DCs) are the professional antigen-presenting cells and dominate this balance of immunogenicity and tolerance. Progesterone (P4) is highlighted as the “hormone of pregnancy” in most eutherian mammals because of its regulatory role in immune-endocrine behavior during pregnancy. Recent studies have shown that P4 is associated with the differentiation and function of DCs, however, the underlying mechanisms remain unidentified. In addition, while progress in the field of immunometabolism has highlighted the intimate connections between the metabolism phenotype and the immunogenic or tolerogenic fate of DCs, whether P4 can affect DCs metabolism and thus exert a functional manipulation has not yet been explored. In this study, we acquired human peripheral blood monocyte-derived DCs and conducted RNA sequencing (RNA-seq) on immature DCs (iDCs), P4-treated DCs (pDCs), and mature DCs (mDCs), aiming to comprehensively assess the effects of P4 on DCs. Our results showed pDCs performed a distinct differentially expressed genes (DEGs) profile compared with iDCs or mDCs. Further functional enrichment and weighted gene co-expression network (WGCNA) analysis found that these DEGs were related not only to the cellular components but also to the significant metabolic activities, including mitochondrial oxidative phosphorylation (OXPHOS) and fatty acid metabolism. In addition, these changes may be involved in the activation of various signaling pathways of PI3K/Akt/mTOR, AMPK/PGC1-α, and PPAR-γ. In summary, our work suggested that P4 induced profound metabolic alterations of mitochondrial OXPHOS and fatty acid metabolism in DCs. Our findings may provide new insights into the role of P4 in DCs.

## Introduction

Pregnancy is a complex and highly coordinated event. Immunologically, the embryo is a semi‐allograft that resides in an immune-competent mother, however, the distinct tolerable microenvironment at the maternal interface provides a guarantee for a successful pregnancy. The underlying mechanisms of maternal-fetal tolerance are multiple including the change of immune cells populations, induction of regulatory T cells, and the shift of Th1 pro-inflammatory to Th2 anti-inflammatory cytokine responses (1, 2). These alternations are sophisticatedly orchestrated under the immune-endocrine interactions. In particular, the female steroid hormone−progesterone (P4), is considered to occupy an important regulatory position in mediating immune response and maintaining pregnancy in humans (3).

P4 is mainly produced by the corpus luteum of the ovary in the menstrual cycle, and this function is subsequently taken over by the placenta after 8 weeks in pregnancy (4, 5). P4 is regarded as the “hormone of pregnancy” and its serum levels undergo a profound change ranging from 10^-9^ M to 10^-6^ M during pregnancy (6). P4 directly regulates gene transcription by binding specific receptors (7). Many studies have reported that P4 is widely involved in endometrial spiral artery remodeling, trophoblast cells adhesion, proliferation, and stromal cells decidualization (8, 9). In addition, studies have emphasized P4 as a communication bridge of endocrine-immune to mediate maternal tolerance to the fetus *via* acting on a series of decidual immune cells, including natural killer cells, T cells, and dendritic cells (DCs) (10–13). Remarkably, the effect of P4 on DCs has gained great attention because of its plastic talent in inducing antigen-specific immunity or tolerance at the maternal-fetal interface.

DCs are originally described as the most potent antigen-presenting cell, and play a central role in both innate and adaptive immunity, despite their small proportions. DCs are divided into two different developmental stages: immature DCs (iDCs) and mature DCs (mDCs) (14). iDCs are usually found to have a limited migratory and T cell priming capacity and serve as “surveillance police” to induce immune tolerance, whereas mDCs exhibit an exceptional ability for antigen presentation and T cell activation and therefore triggers a strong immune response (15). Moreover, iDCs can differentiate toward immunogenic mDCs when activated by various antigens *in vivo* or typical toll-like receptor (TLR) agonists such as lipopolysaccharide (LPS) *in vitro* (16). DCs are not only positively regulated for their maturation as described above, but also negatively responded to signals that prevent their activation, such as dexamethasone and vitamin D (17, 18). Recently, some studies reported that P4 can suppress the production of pro-inflammatory cytokines TNF-α and IL-1β, and reduce CD40 and CD80 expression in DCs (19–21). These studies emphasized that DCs were highly responsive to P4 and can be modified in terms of their activities and functions. However, the number of studies on this topic is still limited, and the underlying mechanism remains poorly understood.

Immunometabolism, an emerging field that explores how metabolism affects immune cell functions, has been well understood in the last decade (22, 23). Numerous studies have revealed that DCs performed preferred metabolism pathways in activated or resting status (24–26). Moreover, metabolism can also directly determine the immunogenicity or tolerability of DCs (27, 28). Interestingly, a recent study demonstrated that P4 can activate hypoxia-inducible factor 1α (HIF-1α) and c-Myc signal to maintain aerobic glycolysis and decidualization in decidualized cells (29). Therefore, it is reasonable to speculate that the metabolism phenotype of DCs may be regulated by P4. In this study, we aimed to determine the metabolic changes and key modules genes underlying human peripheral blood monocyte-derived DCs treated with P4 (pDCs) by RNA sequencing (RNA-seq) and weighted gene co-expression network analysis (WGCNA). Functional enrichment analysis was further applied to help in identifying the most important candidate genes involved in metabolism for quantitative real-time polymerase chain reaction (qRT-PCR) and immunoblot validation.

## Materials & Methods

### *In Vitro* Generation and Treatment of Human DCs

**Figure 1:**
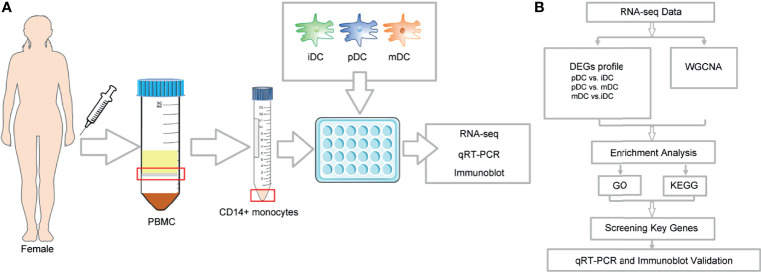
Illustration of the study design framework and RNA-seq analysis flow.

This study was approved by the Institutional Review Board of Reproductive Research Ethics Committees of Shenzhen Zhongshan Urology Hospital (Approval number: SZZSECHU-2020005). Informed consent was obtained following the Declaration of Helsinki. A protocol for the generation of human peripheral blood monocyte-derived DCs was based on previous research with some minor modifications (30). In brief, human monocytes were isolated from peripheral blood mononuclear cells (PBMCs) of healthy women volunteers by density centrifugation at 300 g for 10 minutes (Ficoll–Paque, GE Healthcare) and were purified by CD14 MicroBeads (Miltenyi Biotec, Bergisch Gladbach, Germany, purity >95%) according to the manufacturer’s instructions. Then the cells were cultured in 24-well plates (Corning, China) with RPMI 1640 (Gibco, USA), supplemented with 10% FBS (Gibco, USA), 100 U/ml penicillin, and 100mg/ml streptomycin placed in an incubator under 5% CO_2_ at 37°C. 10 ng/mL recombinant human IL-4 (R&D Systems) and 20 ng/mL recombinant human granulocyte-macrophage colony-stimulating factor (GM-CSF) (R&D Systems) were also added to induce the differentiation of the cells. Refreshment of the culture medium and cytokines was performed on day 2. To obtain P4-induced DCs, cells were treated on day 4 with 10^-8^ M P4 (Sigma-Aldrich). Then cells were either treated with 1μg/mL LPS (Sigma-Aldrich) on day 5 or left untreated. On day 7, immature, mature, and P4-treated DCs were photographed with a High Content Analysis System (Operetta CLS, PerkinElmer) before being harvested for further experiments. The whole process was displayed in **Figure 1A**.

### RNA Collection and Sequencing

Total RNA was isolated using RNA Purification Kit (Thermo Fisher) following the instructions of the manufacturer, and the purity and integrity were checked using the NanoPhotometer^®^ spectrophotometer (IMPLEN, CA, USA) and RNA Nano 6000 Assay Kit of the Bioanalyzer 2100 system (Agilent Technologies, CA, USA), respectively. Subsequently, a total amount of 3 µg RNA was used for the RNA sample preparations. First-strand and second-strand cDNA were synthesized according to the manufacturer of M-MuLV Reverse Transcriptase (RNase H^–^), and DNA Polymerase I and RNase H. cDNA fragments of 250∼300 bp in length were selected and purified with the AMPure XP system (Beckman Coulter, Beverly, MA, USA). Then 3 µl USER Enzyme (NEB, USA) was used with cDNA at 37°C for 15 min followed by 5 min at 95°C before PCR and products were purified with AMPure XP system. Library quality was assessed on the Agilent Bioanalyzer 2100 system. The clustering of the index-coded samples was performed on a cBot Cluster Generation System using TruSeq PE Cluster Kit v3-cBot-HS (Illumia) according to the manufacturer’s instructions. After cluster generation, the library preparations were sequenced on an Illumina Hiseq platform, and 125 bp/150 bp paired-end reads were generated. RNA-seq data have been deposited to NCBI’s Sequence Read Archive with accession number PRJNA777391 (available at: https://www.ncbi.nlm.nih.gov/sra/). Finally, the overall workflow of data analysis was shown in **Figure 1B**.

### Differentially Expressed Genes (DEGs) Analysis

The DESeq2 R package (1.16.1) was applied to perform DEGs analysis among iDCs, pDCs, and mDCs. The *P*-value was adjusted using Benjamini and Hochberg’s approach for controlling the false discovery rate. Genes with an adjusted *P*-value of <0.05 were assigned as differentially expressed. The Venn diagram was drawn by using the online Venn diagrams tool (https://bioinfogp.cnb.csic.es/tools/venny/index.html) and volcano plots were represented with the R packages (1.16.1).

### Gene Ontology (GO) and Kyoto Encyclopedia of Genes and Genomes (KEGG) Enrichment Analysis

GO and KEGG enrichment analysis of DEGs were implemented by the cluster profile R package. The GO terms consist of the following three parts: biological process (BP), cell component (CC), and molecular function (MF). GO and KEGG terms with a corrected *P-*value of less than 0.05 were considered significantly enriched by DEGs.

### WGCNA Analysis

WGCNA is a method for analyzing gene expression patterns of multiple samples, which can cluster genes with similar expression patterns and analyze the relationship between modules and specific traits or phenotypes. The genes with median absolute deviation (MAD) ≤ 0.01 were filtered, and the soft-power threshold of β = 13 was selected. WGCNA analysis was performed in R with the WGCNA package. The further GO and KEGG enrichment was performed using Metascape.

### RNA Collection and qRT-PCR Analysis

Total RNA was acquired as above described. 500 ng total RNA was used to synthesize cDNA using the PrimeScript™ RT Reagent Kit with gDNA Eraser (Takara, Japan) with the Bio-Rad system. Synthesized cDNA was carried for qRT-PCR in a 10 μl reaction using Luna^®^ Universal qPCR Master Mix (New England Biolabs) with primers specific to the genes. The reaction was performed in the QuantStudio 5 Real-Time PCR system (Applied Biosystems), following the manufacturer’s instructions. The 2^−ΔΔ Ct^ method was determined to calculate and quantify the gene expression. All mRNA levels were normalized to β-actin. Primers were designed with computer assistance based on GeneBank, and the sequences were listed in **Table 1**.

**Table 1:** Human forward and reverse primers for validation by qRT-PCR.

Primers	Forward primer (5′-3′)	Reverse primer (5′-3′)
MPC1	ATTTGCCTACAAGGTACAGCC	AGTCATCTCGTGTTTGATAAGCC
SDHC	TAGGTTCAAACCGTCCTCTGT	GAGAGACCCCTGCACTCAAAG
SDHB	TGAATAAGTGCGGACCTATGGTGTTG	GAGCCACAGATGCCTTCTCTACAAG
UQCRQ	CGCGAGTTTGGGAATCTGAC	TAGTGAAGACGTGCGGATAGG
ATP5A	ATGACGACTTATCCAAACAGGC	CGGGAGTGTAGGTAGAACACAT
COX II	CCATCCCTACGCATCCTTTAC	GTTTGCTCCACAGATTTCAGAG
NDUFB8	ACAGGAACCGTGTGGATACAT	CCCCACCCAGCACATGAAT
FASN	TCGTGGGCTACAGCATGGT	GCCCTCTGAAGTCGAAGAAGAA
ACACA	CATCAAATGCATCAGCAGAGACT	CTGCGTCATATGGATGATGGAAT
HADHA	AAATTGACAGCGTATGCCATGA	GCTTTCGCACTTTTTCTTCCACT
HADHB	CTGTCCAGACCAAAACGAAGAA	CGATGCAACAAACCCGTAAGC
CPT2	CTGGAGCCAGAAGTGTTCCAC	AGGCACAAAGCGTATGAGTCT
CPT1	TCCAGTTGGCTTATCGTGGTG	TCCAGAGTCCGATTGATTTTTGC
PI3K	AAGAAGCAAGCAGCTGAG	CTACAGAGCAGGCATAG
AKT	GCCTTTGCCGATCCGC	GCCGTAGCCGTTGTCG
mTOR	ATGACGAGACCCAGGCTAA	GCCAGTCCTCTACAATACGC
HIF-1α	GAAAGCGCAAGTCTTCAAAG	TGGGTAGGAGATGGAGATGC
AMPK	GGCACGCCATACCCTTGAT	TCTTCCTTCGTACACGCAAATAA
PGC1-α	TCTGAGTCTGTATGGAGTGACAT	CCAAGTCGTTCACATCTAGTTCA
PPAR-γ	CGGTGACTTATCCTGTGGTCC	CCGCAGATTCTACATTCGATGTT
β-actin	TCGTGCGTGACATTAAGGAG	GTCAGGCAGCTCGTAGCTCT

MPC1, mitochondrial pyruvate carrier 1; SDHB, succinate dehydrogenase complex iron sulfur subunit B; SDHC, succinate dehydrogenase subunit C; UQCRQ, ubiquinol-cytochrome c reductase complex III subunit VII; ATP5A, ATP synthase F1 subunit alpha; COX II, cytochrome c oxidase subunit II; NDUFB8, NADH ubiquinone oxidoreductase subunit B8; FASN, fatty acid synthase; ACACA, acetyl-CoA carboxylase-1; HADHA, hydroxyacyl-CoA dehydrogenase trifunctional multienzyme complex subunit alpha; HADHB, hydroxyacyl-CoA dehydrogenase trifunctional multienzyme complex subunit beta; CPT2, carnitine palmitoyltransferase II; CPT1, carnitine palmitoyltransferase I; PI3K, phosphoinositide-3-kinase; AKT, protein kinase B; mTOR, mammalian target of rapamycin; HIF-1α, hypoxia-inducible factor 1α; AMPK, Adenosine 5-monophosphate-activated protein kinase; PPAR-γ, peroxisome proliferator activated receptor gamma; PGC1-α, PPAR-γ coactivator 1 alpha; qRT-PCR, quantitative real-time polymerase chain reaction.

### Immunoblot Analysis

Cells were harvested and lysed with radioimmunoprecipitation (RIPA; Beyotime, Shanghai, China) lysis buffer, and the lysates were centrifuged at 4°C 12000g for 15 min to collect the supernatant. The BCA assay kit (Beyotime, Shanghai, China), was used to quantify protein concentrations. An automated Wes Capillary System (Protein Simple, San Jose, CA, 12-230 kDa kit cat. SM-W004) was used to detect the proteins levels of oxidative phosphorylation (OXPHOS) according to the manufacturer’s protocol. Total protein loading was 0.75 μg/μl. Antibody signals were determined to be saturated at 1:50 for Total OXPHOS Human WB Antibody Cocktail (Abcam, ab110411) and 1:200 for vinculin antibody (Abcam, ab129002). Immunoblot analysis was executed using the Compass for Simple Western Program (Protein Simple).

### Statistical Analysis

Continuous data with normal distribution were presented as the mean ± standard error of mean (SEM), and analyzed by analysis of variance (ANOVA) using SPSS 22.0 (IBM SPSS, USA). Continuous variables without normal distribution were presented as median and interquartile range and were analyzed by Kruskal-Wallis test, pairwise comparison. All *P-*values were two-tailed and significance was set as ^*^ *P-*value <0.05, ^**^ *P-*value <0.01. All experiments were independently repeated at least three times.

## Results

### Morphological Characterization of iDCs, pDCs, and mDCs

**Figure 2:**
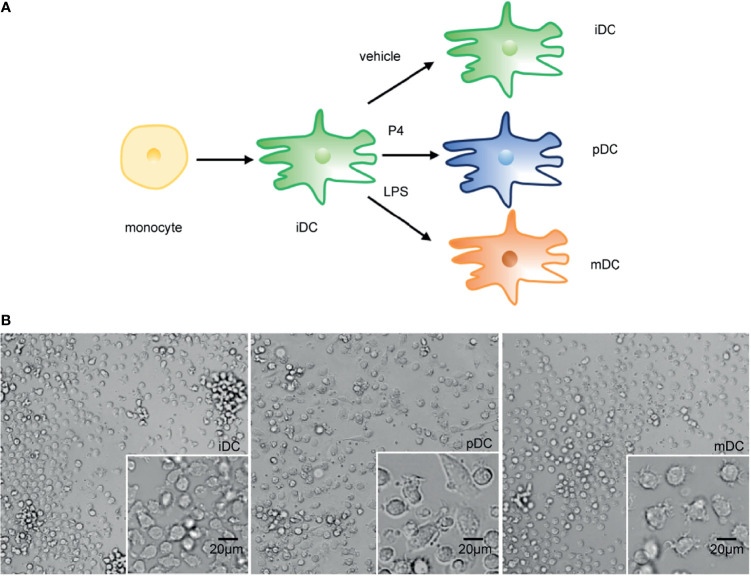
Schematic diagram of DCs treatments and representative morphological pictures. **(A)** Schematic diagram of DCs treatments. **(B)** Representative monolayer cells pictures of iDC, pDC, and mDC on day 7. Magnification, x20; x40. Scale bar = 20 μm. P4, progesterone; LPS, lipopolysaccharide; iDC, immature DC; pDC, P4-treated DC; mDC, mature DC.

To investigate the alterations of human DCs exposed to P4, the morphological features of iDCs, pDCs, and mDCs were observed and photographed on day 7. Compared to the round-shaped iDCs, the mDCs showed more small dendrites, as previously reported (30). pDCs presented a completely different morphological feature from mDCs. These cells became larger with spindle-shaped dendrites (**Figure 2**). Moreover, all DCs appeared to be a typical characterization of the cell colony. The result showed that DCs developed a distinct morphological change after P4 treatment.

### DEGs Identification of iDCs, pDCs, and mDCs

**Figure 3:**
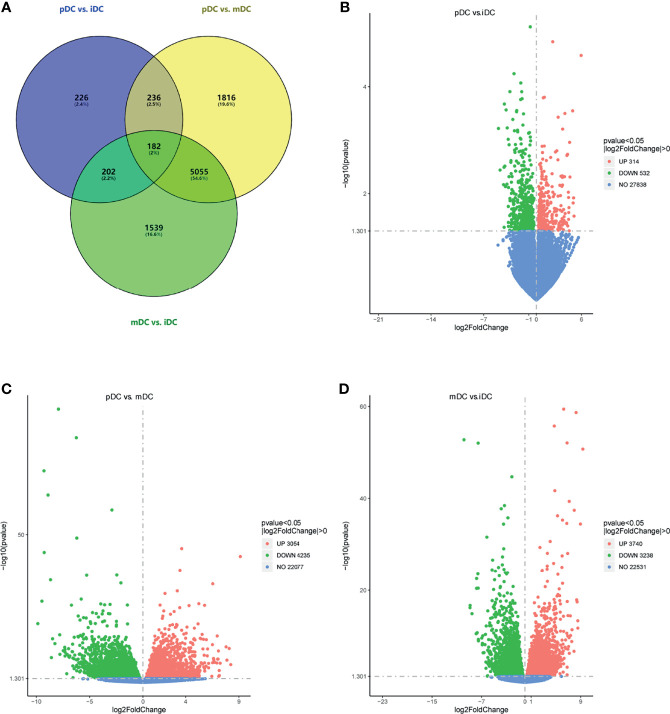
DEGs analysis. **(A)** Venn diagram of DEGs (pDC vs. iDC, pDC vs. mDC, and mDC vs. iDC). **(B–D)** Volcano map of DEGs (pDC vs. iDC; pDC vs. mDC, and mDC vs. iDC, respectively). Red dots indicated upregulated DEGs, green dots indicated downregulated DEGs, and blue dots indicated genes that were not differentially expressed. DEGs, differentially expressed genes; iDC, immature DC; pDC, P4-treated DC; mDC, mature DC.

To investigate the effects of P4 on DCs, 5 samples in the iDCs, pDCs, and mDCs group were selected for gene sequencing, respectively. Genes with an adjusted *P*-value <0.05 were categorized as differentially expressed. The overall distribution of DEGs was shown in a Venn diagram **(** **Figure 3A** **)**. After normalization, P4 induced 846 genes alterations, with 314 upregulated genes and 532 downregulated genes compared with iDCs **(** **Figure 3B** **)**. Comparatively, pDCs showed a total of 7289 DEGs, which contain 3054 upregulated and 4235 downregulated genes versus mDCs (**Figure 3C**). We also detected 6978 differential genes between iDCs and mDCs, with 3740 upregulated and 3238 downregulated genes (**Figure 3D**). These results revealed that P4-treated DCs behaved a distinct gene expression profile, especially when compared with mDCs.

### GO and KEGG Enrichment Analysis

**Figure 4:**
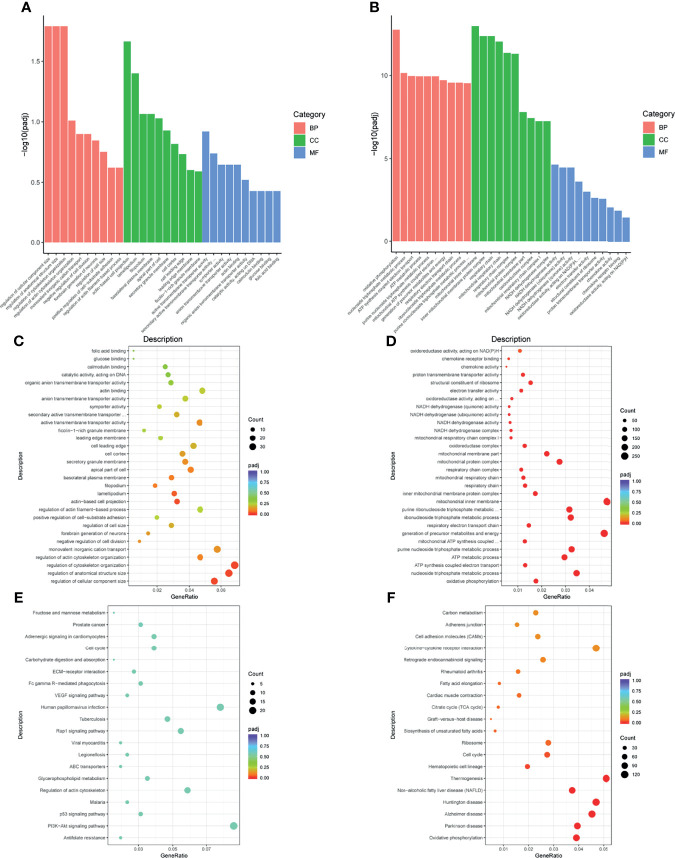
GO and KEGG enrichment analysis. **(A, B)** Bars diagram of GO enrichment analysis (pDC vs. iDC and pDC vs. mDC, respectively). The X-axis denoted detailed annotation classes of GO ontologies by different colors and the Y-axis denoted the percentage of genes. **(C, D)** Dots diagram of GO enrichment (pDC vs. iDC and pDC vs. mDC, respectively). **(E, F)** Dots diagram of KEGG enrichment (pDC vs. iDC and pDC vs. mDC, respectively). The X-axis indicated the proportion of DEGs annotated to GO or KEGG terms to all GO or KEGG annotated DEGs and the Y-axis represented detailed classification of GO or KEGG. Size and color of dots represented the percentage of genes and significance level of adjusted *P*-value, respectively. GO, Gene Ontology; KEGG, Kyoto Encyclopedia of Genes and Genomes; BP, biological process; CC, cellular component; MF, molecular function; DEGs, differentially expressed genes; iDC, immature DC; pDC, P4-treated DC; mDC, mature DC.

To recognize the feature and function of these DEGs in detail, we conducted GO and KEGG enrichment analysis by the cluster Profiler R package, in which gene length bias was corrected. GO terms with a corrected *P*-value of less than 0.05 were considered to be significantly enriched. Based on this screening criterion, we identified the most significant GO annotation, which consists of biological process (BP), cell composition (CC), and molecular function (MF). Compared to iDCs, DEGs in pDCs were mainly enriched in the regulation of cellular components size, cytoskeleton organization, actin-based cell projection (**Figure 4A**). When compared with mDCs, pDCs showed a significant gene enrichment associated with metabolic behaviors, including OXPHOS, mitochondrial protein complex, mitochondrial respiratory chain, and NADH dehydrogenase activity (**Figure 4B**). These GO terms were also illustrated as dot plots, with the gene ratio denoted by size, and the significance denoted by color (**Figures 4C, D**). The detailed gene information of the top 10 GO annotations was also displayed in **Table 2**. To further investigate the potential pathway involved in these DEGs, we also selected the top 20 of KEGG enrichment results. We found that pDCs were mainly enriched in the regulation of actin cytoskeleton, phosphoinositide-3-kinase (PI3K)/protein kinase B (Akt) signaling, and glycerophospholipid metabolism compared with iDCs (**Figure 4E**). Interestingly, when compared with mDCs, pDCs presented significant enrichment involved in metabolic activities, including OXPHOS, citrate cycle, fatty acid elongation, and biosynthesis of unsaturated fatty acids (**Figure 4F**). We also listed the detailed gene information related to these metabolic pathways in **Table 3**. In addition, we observed several canonical metabolism‐related signaling, including the peroxisome proliferator activated receptor (PPAR) signaling, mammalian target of rapamycin (mTOR) signaling, HIF-1α signaling, and adenosine 5-monophosphate-activated protein kinase (AMPK) signaling although the *P*-values were slightly above the 0.05 significance threshold (**Table S1**). Collectively, these data implied that compared with iDCs or mDCs, the DEGs of pDCs were associated with the cellular cytoskeleton and cellular metabolic events including OXPHOS and fatty acid metabolism.

**Table 2:** Top 10 enriched GO terms.

Category	Description	*p*-value	Gene name
CC	Mitochondrial inner membrane	1.1E-13	ATP5MF/UQCR10/NDUFA2/COX5B/ATP5PB
			HADHB/UQCRC2/MPC1/UQCR11/HADHA
			COX18/SDHD/CPT2/MPC2/SDHB
BP	Oxidative phosphorylation	1.8E-13	NDUFB5/UQCR10/NDUFA2/COX5B/ATP5PB
			NDUFA1/UQCRC2/COX7C/NDUFB6/UQCRQ
			UQCRC1/COX6C/NDUFB9/COX5A/CYC1
CC	Inner mitochondrial membrane protein complex	4.3E-13	ATP5MF/UQCRFS1/NDUFA4/NDUFB2/UQCR10
			NDUFA2/COX5B/ATP5PB/NDUFA1/UQCRC2
			NDUFB6/UQCRQ/SDHC/ATP5F1B/ATP5MD
CC	Respiratory chain	4.3E-13	NDUFS4/SDHC/UQCR11/NDUFS5/NDUFS6
			NDUFA3/NDUFB8/MTCO2/NDUFB10/NDUFV1
			NDUFA10/NDUFS8/SDHAF4/COX7A1/SDHB
CC	Respiratory chain complex	4.4E-12	NDUFB5/UQCR10/COX7C/NDUFA8/COX8A
			NDUFA9/NDUFAB1/COX4I1/COX7B/NDUFS3
			NDUFA5/SDHD/NDUFA12/SDHB/UQCRHL
CC	Mitochondrial protein complex	5E-12	UQCR10/NDUFA2/COX5B/ATP5PB/NDUFA1
			TIMM13/UQCRC2/COXII/COX7C/NDUFS4
			SDHC/ATP5F1B/ATP5MD/ATP5MPL/HADHA
BP	ATP metabolic process	1.1E-10	UQCRQ/NDUFB3/ATP5F1A/SLC25A33/UQCRH
			ENPP1/COX6B1/SDHC/COX4I1/LDHA
			ATP5PF/PKM/HK3/COX5A/HIF1A
BP	Mitochondrial ATP synthesis coupled electron transport	1.1E-10	COX4I1/COX7B/NDUFS3/NDUFA5/SDHD
			MT-ND4L/COX6C/SDHAF2/NDUFB8/MT-ND5
			NDUFB7/CCNB1/CDK1/NDUFB1/NDUFC1
BP	Generation of precursor metabolites and energy	1.9E-10	COX8A/NDUFA9/ATP5ME/SDHD/DDIT4
			ATP5F1C/MDH2/NUP35/COA6/HIF1A
			AOC2/DHA1/GPD1/PFKFB1/HK2
BP	Electron transport chain	4.7E-10	UQCRC2/COX7C/SDHD/AKR7A2/TSTA3
			GPD2/NDUFB11/NDUFC2/TXNRD2/HSD17B6
			COX7A2L/UGDH/COX7A1/HAAO/NDUFAF1

GO, Gene Ontology; BP, biological process; CC, cellular component; MF, molecular function.

**Table 3:** Most significant KEGG metabolism pathways.

Terms	Description	*p-*value	Gene name
hsa00190	Oxidative phosphorylation	5.2E-38	ATP5MF/UQCRFS1/NDUFA4/NDUFB2/ATP5MC1/NDUFV3
			NDUFB5/UQCR10/NDUFA2/COX5B/ATP5PB/NDUFA1
			UQCRC2/COX7C/NDUFA8/ATP5MC3/COX8A/NDUFA9
			ATP5ME/UQCRB/ATP5F1D/NDUFB6/UQCRQ/NDUFB3
			ATP5F1A/UQCRC1/PPA2/COX7A2/UQCRH/COX6B1
			NDUFS7/NDUFS4/SDHC/ATP5F1B/ATP6V1F/UQCR11
			NDUFS5/NDUFAB1/ATP6V1D/COX4I1/COX7B/ATP5MG
			MTATP8/NDUFS3/ATP5MC2/NDUFA5/ATP6V0E1/SDHD
			ATP5F1C/ATP5F1E/MTND4L/COX6C/NDUFB9/COX17
			ATP5PF/NDUFB4/COX5A/CYC1/ATP6V0D1/NDUFS6
			NDUFA3/NDUFB8/MTND5/NDUFB7/NDUFB1/NDUFC1
			MTCO2/NDUFB10/NDUFV1/ATP6V1G1/NDUFA10
			NDUFS8/MTCO1/NDUFS1/ATP6V0A1/NDUFB11
			NDUFC2/ATP6V0B/ATP6V1B2/COX7A2L/COX7A1
			ATP5PD/ATP6V1E1/ATP6V1A/NDUFA12/SDHB
			UQCRHL/NDUFA6/ATP5PO/COX6A1
hsa00020	Citrate cycle	2.82E-06	IDH1/PC/MDH1/PDHB/SDHC/IDH3G/SUCLG1
			SDHD/MDH2/FH/ACO2/PDHA1/IDH2/SUCLG2
			SUCLA2/SDHB/IDH3B
hsa01040	Biosynthesis of unsaturated fatty acids	6.81E-05	SCD/FADS2/ACOT7/FADS1/HADHA/TECR
			ELOVL5/ACAA1/ACOT1/ACOT4/ACOX1
			HSD17B12/ACOT2/HACD1
hsa00062	Fatty acid elongation	0.00472	ECHS1/ACAA2/HADHB/ACOT7/PPT1/HADHA
			ABHD17A/TECR/ELOVL5/ACOT1/ACOT4/HSD17B12
			ACOT2/HACD1

KEGG, Kyoto Encyclopedia of Genes and Genomes.

### WGCNA Analysis

**Figure 5:**
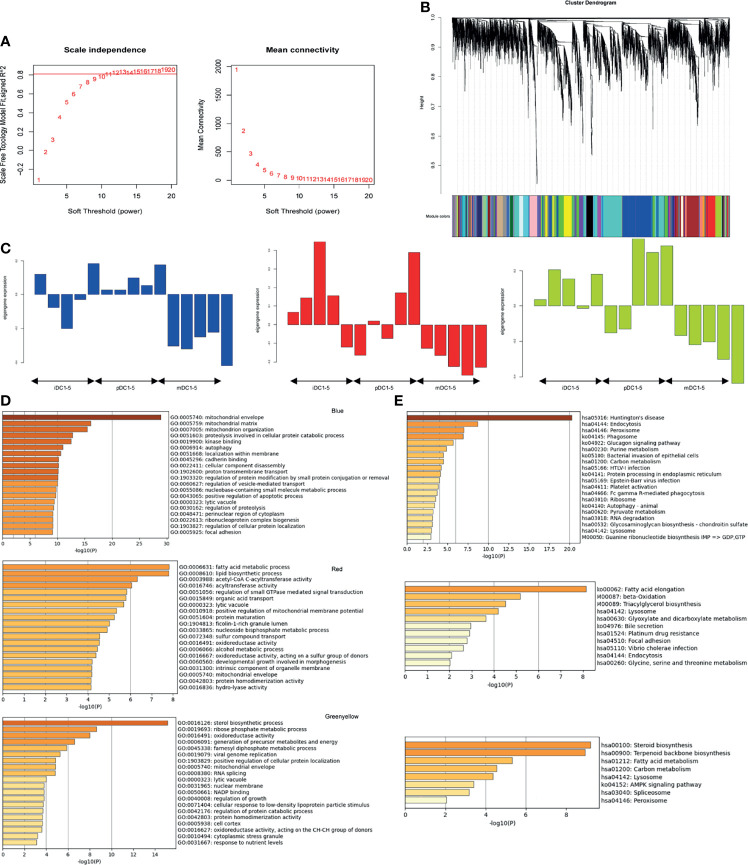
WGCNA analysis. **(A)** Determination of soft-threshold power. **(B)** Cluster dendrogram of genes. **(C)** Expression pattern of the genes and eigengenes in the blue, red, and green-yellow modules. **(D)** GO enrichment results for genes in blue, red, and green-yellow modules. **(E)** KEGG enrichment results for genes in blue, red, and green-yellow modules. GO, Gene Ontology; KEGG, Kyoto encyclopedia of genes and genomes; iDC, immature DC; pDC, P4-treated DC; mDC, mature DC.

To further explore the changes that DCs respond to P4, we also conducted a WGCNA analysis. A scale-free co-expression network was constructed with a soft threshold of 13 (**Figure 5A**). Genes in the same module had similar expression patterns in different samples, and modules were distinguished by different colors. A total of 26 modules were generated, of which the blue, red, and green-yellow modules showed the highest correlation and were selected for further analysis (**Figures 5B, C**). Our enrichment results showed that GO annotations in the red module were mainly related to fatty acid metabolism, lipid biosynthetic process, acetyl-CoA, and C-acyltransferase activity. The blue and green-yellow modules were enriched in mitochondrial biology, oxidoreductase activity, and the generation of cellular metabolites and energy (**Figure 5D**). Moreover, KEGG analysis of these three modules exhibited enrichment in metabolic activities, such as fatty acid elongation or oxidation (**Figure 5E**). These data suggested that P4 treatment induced the fatty acid metabolism and mitochondrial alterations of DCs.

### Validation of qRT-PCR and Immunoblot

**Figure 6:**
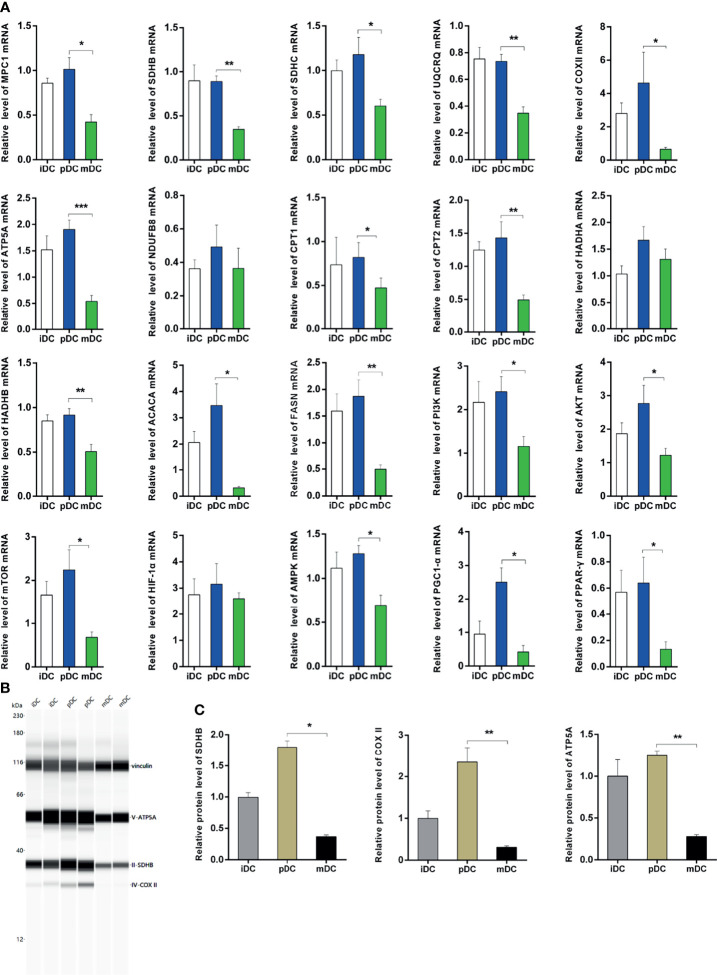
Validation of qRT-PCR and immunoblot. **(A)** Expression of genes associated with OXPHOS, FAO, and FAS using qRT-PCR. All values were normalized against the mRNA expression level of β-actin. **(B, C)** Protein levels and quantification of OXPHOS. qRT-PCR, quantitative real-time polymerase chain reaction; OXPHOS, oxidative phosphorylation; FAO, fatty acid oxidation; FAS, fatty acid synthesis; iDC, immature DC; pDC, P4-treated DC; mDC, mature DC. *P < 0.05, **P < 0.01, ***P < 0.001.

To validate the RNA-seq data, we selected 20 genes related to the metabolism of all DEGs to perform qRT-PCR. Our results showed that the genes of mitochondrial complexes II-V (succinate dehydrogenase complex iron sulfur subunit B, SDHB; succinate dehydrogenase complex subunit C, SDHC; ubiquinol-cytochrome C reductase complex III subunit VII, UQCRQ; cytochrome c oxidase subunit II, COX II, and ATP synthase F1 subunit alpha, ATP5A) were highly expressed in pDCs compared with mDCs, though no significant differences were detected in complex I− NADH ubiquinone oxidoreductase subunit B8 (NDUFB8) (**Figure 6A**). These complexes are important components of the electron transport chain and the oxygen-coupled ATP synthesis in the mitochondrial OXPHOS process. Our data showed that the mRNA levels of mitochondrial pyruvate carrier 1 (MPC1) were upregulated in pDCs compared with mDCs, which is a key factor for mitochondrial pyruvate carrier and sequent OXPHOS (**Figure 6A**). We also demonstrated significantly increased protein levels of OXPHOS complexes in pDCs compared with mDCs (**Figures 6B, C**). Additionally, we found that hydroxyacyl-CoA dehydrogenase trifunctional multienzyme complex subunit beta (HADHB), carnitine palmitoyltransferase I (CPT1), and II (CPT2), which are necessary for fatty acid oxidation (FAO), and acetyl-CoA carboxylase alpha (ACACA) and fatty acid synthase (FASN), which are involved in the fatty acid synthesis (FAS), were also increased in mRNA levels in pDCs compared with mDCs (**Figure 6A**). Moreover, we observed upregulated mRNA expression of AMPK, PPAR-γ coactivator 1 alpha (PGC1-α), PI3K, Akt, mTOR, and PPAR-γ in pDCs compared with mDCs, whereas the expression of HIF-1α had no change (**Figure 6A**). Taken together, these findings suggested DCs present a distinct phenotype following P4 treatment with general trends in association with enhanced OXPHOS and fatty metabolic activities.

## Discussion

P4 has long been considered as the master hormone of pregnancy. In recent years, P4 is put forward as a potential medium for regulating the maternal immune system *in vivo* and for maintaining maternal-fetal immune tolerance (13, 31, 32). These effects are achieved through interaction with a series of decidual immune cells, such as T cells, natural killer cells, macrophages, and DCs. Although some profound findings demonstrated the influence of P4 on lymphocytes, there are limited studies exploring the effects of P4 on DCs functions, and the mechanism underlying these effects (19, 33–35). DCs perform an extraordinary capacity to balance immunogenicity and tolerance for providing a protected environment during normal pregnancy. Therefore, it is interesting to explore the role of P4 on DCs. In the present study, we obtained an information-rich gene expression profile of human DCs treated with P4 by RNA-seq. Further analysis showed that P4 dramatically altered the expression of key genes and pathways involved in the metabolism of DCs, which may be a promising manipulator in modulating DCs tolerance. This current study provided the first evidence that P4 regulated the metabolism of DCs and helped to further improve our understanding of the effect of P4 on DCs.

In this study, we firstly analyzed the treatment and the corresponding gene data of DCs and found pDCs behaved a significant different DEGs profile from that of iDCs or mDCs. Compared to iDCs, the GO enrichment analysis in pDCs revealed enriched terms related to cellular components and cytoskeleton. Interestingly, the mitochondria were identified as the core of GO cellular component terms in pDCs, such as mitochondrial protein complex, mitochondrial inner membrane, mitochondrial respiratory chain, and electron transfer activity compared with mDCs. The mitochondrial respiratory chain, also known as the mitochondrial electron transport chain, consists of five enzyme complexes (complexes I-V) situated in the inner mitochondrial membrane that couple oxidation to phosphorylation, and provide ATP. KEGG analysis also showed that DEGs in pDCs were primarily concentrated in the OXPHOS process and fatty acid metabolism. These data suggested that P4 induced obvious changes in gene transcription levels that point to mitochondrial metabolism of DCs. Our qRT-PCR validation demonstrated that P4 upregulated the mRNA levels of mitochondrial complexes II-V (SDHB, SDHC, UQCRQ, ATP5A, COX II) in DCs. We also further confirmed increased protein levels of OXPHOS complexes, including SDHB, COX II, and ATP5A. In addition, we found upregulated mRNA levels of MPC1, which is essential for mitochondrial pyruvate transport and subsequent OXPHOS process. These findings together indicated that human DCs initiated early OXPHOS metabolic pathways at the mRNA and protein levels after exposure to P4. According to WGCNA results and literature research, we also selected genes related to FAO (CPT1, CPT2, HADHB) and FAS (FASN, ACACA) for validation, and the results showed that these genes were significantly upregulated in pDCs compared with mDCs. In detail, FAO involves two key steps of fatty acid transfer and β-oxidation. Both CPT1 and CPT2 are crucial to transfer activated long-chain fatty acids into mitochondria while HADHB acts in the subsequent β-oxidation process as one of the mitochondrial thiolates. In summary, our study identified profound mitochondrial and fatty metabolic alterations in human DCs regulated by P4.

It has long been acknowledged that the activation and function of immune cells correlate with, and are supported by alterations in their metabolic pathways (22, 23). Phenomena accompanying immunological responses involve changes in gene expression that are thermodynamically demanding, requiring fast metabolic adaptations, which is known as immunometabolism. Accumulating evidence has indicated that the metabolic programming of immune cells is an important determinant of their function (36, 37). DCs were reported to undergo metabolic reprogramming in response to multiple environmental factors. To date, the metabolic features of immunogenic DCs have become more well characterized. Krawczyk et al. demonstrated that TLR agonists stimulated a profound metabolic transition to aerobic glycolysis in mouse DCs. This metabolic switch depended on the PI3K/Akt pathway, was antagonized by the AMPK, and was important for DCs maturation and function (38). Induction of DCs glycolysis was also essential for the anabolic demands that underpinned the production of membranes needed for expansion of the endoplasmic reticulum and Golgi to accommodate the stimulation *via* TLR agonists (39). Several studies have reported metabolic characteristics of tolerogenic DCs despite the limitations of the available research. Proteomic analysis of human DCs treated with vitamin D and dexamethasone, two well-known immunosuppressive agents that induced tolerogenic DCs, revealed increased expression of genes associated with mitochondrial metabolism and OXPHOS (17, 30). Malinarich et al. confirmed similar results that human tolerogenic DCs displayed a markedly augmented catabolic pathway, related to high oxidative phosphorylation and fatty acid metabolism levels (40). Another recent study showed that tolerogenic effects of vitamin D on DCs also involved FAS (18). Thus, a current generally accepted opinion shows that increased OXPHOS and FAO are tightly associated with the tolerogenic function of DCs (25, 28, 41, 42). The above findings emphasize that the activation and function of DCs are dictated by the type of metabolism these cells commit to. In our current study, P4-treated DCs performed high OXPHOS and fatty acid metabolism capacities. This may provide promising information that P4 may be another potential tolerogenic medium that coordinates the metabolism and immune tolerogenic function in DCs during pregnancy. These observations also point to a mechanism for rapid genome-wide reprogramming by modulation of underlying cellular metabolism during DC differentiation. Our findings may contribute to the identification of P4 as new metabolic targets for manipulating DC function. This knowledge may be used in the rational design of strategies to improve the immunogenicity or tolerogenic of DCs in clinically relevant settings.

Additionally, we also tentatively demonstrated that P4 upregulated mRNA levels of PI3K, Akt, mTOR, AMPK, PGC1-α, and PPAR-γ in DCs. The mTOR and its upstream PI3K/Akt have been well known as metabolism‐related regulation signaling pathways in DCs (24). AMPK is a central regulator of catabolic metabolism and has been shown to activate the PGC-1α, which promotes mitochondrial biogenesis to increase mitochondrial OXPHOS (43). PPAR-γ, a sensor for fatty acids, was recently confirmed to be involved in the functional regulation of human DCs (27). Taken together, these data suggested that the above metabolic effects in pDCs may be mediated by these signaling pathways, however, further studies are needed to clarify the mechanism.

In summary, our current study revealed a comprehensive RNA-seq analysis and identified some critical molecules and pathways related to metabolism between P4 and DCs. Our results provided the first evidence that P4-treated DCs had a defined metabolic phenotype characterized by a prominent mitochondrial OXPHOS and high fatty acid metabolic phenotype. These data may provide a novel insight into the role of P4 in DCs.

## Data Availability Statement

The datasets presented in this study can be found in online repositories. The names of the repository/repositories and accession number(s) can be found below: https://www.ncbi.nlm.nih.gov/, accession ID: PRJNA777391.

## Ethics Statement

The studies involving human participants were reviewed and approved by The Institutional Review Board of Reproductive Research Ethics Committees of Shenzhen Zhongshan Urology Hospital. The patients/participants provided their written informed consent to participate in this study.

## Author Contributions

YZ, TY, and SL supported the research. SZ and SL conceived the original idea and the structure of the manuscript. SZ performed the experiments and drafted the first version of the manuscript. LH assisted in the experiments and manuscript. XW collected the clinical samples. LD and SC provided critical feedback and helped revise the manuscript. All authors contributed to the article and approved the submitted version.

## Funding

This work was supported by the National Key Research & Developmental Program of China (2018YFC1003900/2018YFC1003904/2018YFC1004601), National Natural Science Foundation of China (No. 82001547), Guangdong Basic and Applied Basic Research Foundation (2019A1515010914), Shenzhen Fundamental Research Program (JCYJ20190813160809117).

## Conflict of Interest

The authors declare that the research was conducted in the absence of any commercial or financial relationships that could be construed as a potential conflict of interest.

The handling Editor declared a past co-authorship with two of the authors LD and YZ.

## Publisher’s Note

All claims expressed in this article are solely those of the authors and do not necessarily represent those of their affiliated organizations, or those of the publisher, the editors and the reviewers. Any product that may be evaluated in this article, or claim that may be made by its manufacturer, is not guaranteed or endorsed by the publisher.
