# Supplementary material for: Identification of Profound Metabolic Alterations in Human Dendritic Cells by Progesterone Through Integrated Bioinformatics Analysis
Source: Front Immunol. 2021 Dec 17;12:806110. doi: 10.3389/fimmu.2021.806110 (PMC8718397; doi:10.3389/fimmu.2021.806110)
Supplement: Supplementary file 1 [file Table_1.docx]

Supplementary Material

**Table S1 Metabolism‐related signaling pathways of KEGG.**

| **Terms** | **Description** | ***p-*value** | **Gene name** |
| --- | --- | --- | --- |
| hsa03320 | PPAR signaling | 0.01572 | FABP4/FABP3/DBI/FABP5/LPL/SCD FADS2/SCP2/RXRA/CD36/CYP27A1 PPARG/PPARD/ACAA1/ACSL3/ACOX2 CPT2/ACOX1/AQP7/ACADM/FABP6 |
| hsa04150 | mTOR signaling | - | LPIN1/LAMTOR5/LAMTOR4/SGK1/LAMTOR1 WNT5B/RPS6KA1/ATP6V1F/SEC13/ATP6V1D LAMTOR2/RPS6KB2/HRAS/MAPKAP1 ATP6V1G1/LAMTOR3/DEPTOR/ATP6V1B2 RHOAEIF4E2/RHEB/ATP6V1E1/SEH1L ATP6V1A/RRAGD/EIF4B/FZD8/CAB39L/FZD3 |
| hsa04152 | AMPK signaling | - | FASN/SCD/FBP1/HMGCR/CCND1/CD36 PFKP/RPS6KB2/PPP2R1A/MLYCD/CFTR PPP2R3A/PPARG/PFKL/RHEB/PRKAG3 ADIPOR2/RAB2A/PRKAG2/RAB14 PPP2R2D/CAB39L |
| hsa04066 | HIF-1 signaling | - | FLT1/PDHB/EGLN3/PGK1/RBX1/LDHA RPS6KB2/HK3/ELOB/PDHA1/ENO1/ELOC CAMK2D/LTBR/EIF4E2/PFKL/MKNK1 |
| hsa04151 | PI3K-Akt signaling | - | SGK1/VEGFB/GNGT2/VWF/FLT1/CCND1 GNB4/RXRA/ITGA3/YWHAH/ITGA11/BAD RPS6KB2/FGFR1/PPP2R1A/PPP2R3A/HRAS GNB2/TNC/HSP90AA1/COL6A1/EIF4E2 LAMB1/LAMA2/RHEB/CDC37/FN1/EIF4B PPP2R2D/YWHAB |

Note: PPAR, peroxisome proliferator activated receptor; mTOR, mammalian target of rapamycin; AMPK, Adenosine 5-monophosphate-activated protein kinase; HIF-1, hypoxia-inducible factor 1; PI3K, phosphoinositide-3-kinase; Akt, protein kinase B
